# Comparing the Use of a Mobile App and a Web-Based Notification Platform for Surveillance of Adverse Events Following Influenza Immunization: Randomized Controlled Trial

**DOI:** 10.2196/39700

**Published:** 2023-05-08

**Authors:** A Brianne Bota, Julie A Bettinger, Shirley Sarfo-Mensah, Jimmy Lopez, David P Smith, Katherine M Atkinson, Cameron Bell, Kim Marty, Mohamed Serhan, David T Zhu, Anne E McCarthy, Kumanan Wilson

**Affiliations:** 1 Clinical Epidemiology Program Ottawa Hospital Research Institute Ottawa, ON Canada; 2 Vaccine Evaluation Center BC Children's Hospital Research Institute Vancouver, BC Canada; 3 Karolinska Institutet Solna Sweden; 4 CanImmunize Inc Ottawa, ON Canada; 5 Department of Social and Behavioral Sciences Yale School of Public Health New Haven, CT United States; 6 Department of Medicine University of Ottawa Ottawa, ON Canada; 7 Bruyère Research Institute Ottawa, ON Canada

**Keywords:** active participant–centered reporting, health technology, adverse event reporting, mobile apps, immunization, vaccine, safety, influenza, campaign, apps, mobile, surveillance, pharmacovigilance

## Abstract

**Background:**

Vaccine safety surveillance is a core component of vaccine pharmacovigilance. In Canada, active, participant-centered vaccine surveillance is available for influenza vaccines and has been used for COVID-19 vaccines.

**Objective:**

The objective of this study is to evaluate the effectiveness and feasibility of using a mobile app for reporting participant-centered seasonal influenza adverse events following immunization (AEFIs) compared to a web-based notification system.

**Methods:**

Participants were randomized to influenza vaccine safety reporting via a mobile app or a web-based notification platform. All participants were invited to complete a user experience survey.

**Results:**

Among the 2408 randomized participants, 1319 (54%) completed their safety survey 1 week after vaccination, with a higher completion rate among the web-based notification platform users (767/1196, 64%) than among mobile app users (552/1212, 45%; *P*<.001). Ease-of-use ratings were high for the web-based notification platform users (99% strongly agree or agree) and 88.8% of them strongly agreed or agreed that the system made reporting AEFIs easier. Web-based notification platform users supported the statement that a web-based notification-only approach would make it easier for public health professionals to detect vaccine safety signals (91.4%, agreed or strongly agreed).

**Conclusions:**

Participants in this study were significantly more likely to respond to a web-based safety survey rather than within a mobile app. These results suggest that mobile apps present an additional barrier for use compared to the web-based notification–only approach.

**Trial Registration:**

ClinicalTrials.gov NCT05794113; https://clinicaltrials.gov/show/NCT05794113

## Introduction

Vaccine safety surveillance is a core component of vaccination programs to monitor the safety of vaccines for health care professionals, policy makers, and the public. The implementation of a vaccine safety surveillance program increases the public confidence in various vaccines that are introduced [1]. Serious adverse events following immunization (AEFIs) are rare but do occur. For this reason, good-quality pharmacovigilance is necessary to detect AEFIs. Digital technology offers the potential to enhance and improve AEFI surveillance.

Vaccine pharmacovigilance in Canada currently involves passive and active surveillance systems that are designed to detect even very rare events in the population of vaccine recipients. However, passive surveillance suffers from underreporting and reporting biases (based on age, severity, and type of vaccine), while active surveillance occurs only for adverse events (AEs) in hospitalized children for specific conditions [2,3]. The detection of safety signals can be slow for either system and does not allow for the calculation of population-based incidence rates.

Participant-centered active vaccine reporting offers a potentially more economical and sustainable mechanism to conduct large-scale pharmacovigilance as it allows for rapid identification of AEFIs with minimal human resource needs. The Canadian National Vaccine Safety (CANVAS) network was established in 2009 to provide enhanced monitoring for pandemic and seasonal influenza vaccines using rapid, web-based active surveillance. CANVAS provides timely influenza vaccine safety information annually, via a web-based survey, collected from over 50,000 adults and parents across Canada. CANVAS includes an unvaccinated control group, which provides a robust approach for conducting rapid evaluations of vaccine safety. 

The use of mobile health (mHealth) has allowed researchers, policy makers, and health care practitioners to reach individuals who are often less accessible, which, in turn, encompasses a broader and more representative sample of the population [4]. mHealth has not only increased accessibility but also provided low-cost health care solutions for various populations [5]. The COVID-19 pandemic and influenza outbreaks disproportionally harm individuals from communities that may be less accessible [6]. The need to reach broader populations is important in vaccine safety research to better improve postmarket surveillance and could potentially be aided through low-cost solutions via mHealth. However, this needs to be evaluated.

mHealth is a rapidly growing field, and the near ubiquity of smartphones presents a unique opportunity to incorporate digital technologies to address public health issues, such as monitoring AEFIs, and facilitate communication between individuals and public health officials. With increased mobile device and app usage, the potential exists to capture, transmit, and monitor postimmunization experiences rapidly using self-reporting and personal mobile devices. For example, Australia has successfully implemented participant-centered digital AEFI reporting using SMS text messaging technology [7]. CANImmunize is a digital immunization tracking solution that could serve as an acceptable platform for digital AEFI reporting [8,9].

In this study, we will evaluate the effectiveness and feasibility of using a mobile app for reporting participant-centered seasonal influenza AEFIs compared to a web-based notification platform.

## Methods

### Study Procedures

A 2-centered randomized controlled trial (RCT) was conducted to evaluate the use of safety reporting via a mobile app compared to safety reporting via web-based CANVAS notifications among individuals receiving the influenza vaccine from October 6 to November 29, 2020, during the seasonal influenza vaccine campaign in Ottawa, Ontario, and Vancouver, British Columbia, Canada. 

Individuals were recruited at the time of receiving their influenza vaccine. Eligibility criteria included the ability to speak English or French, having an active email address and telephone number, and being immunized with the seasonal influenza vaccine.

### Randomization 

After study enrollment, participants were randomized to receive the web-based safety survey either through the mobile app or were emailed a link to the web-based survey using a 4-block randomization design.

### Web-Based Notification Arm

All participants randomized to the web-based notification arm received the following web-based CANVAS notifications [10,11]. Briefly, participants received an email notifying them of their registration in the study. Eight days following their influenza vaccine, participants received an email with the survey link asking them to complete their web-based influenza vaccine safety survey. Participants received a reminder email on day 11 if they did not complete their survey. Further details on CANVAS surveillance and a description of the questionnaire can be found in previous studies [10,12,13].

### Mobile App Arm

Participants randomized to the mobile app arm received an email asking them to download the app and activate their account. Users who did not activate their account after 48 hours received a reminder email. Participants who activated their accounts could spontaneously report an AE through the app and were also notified of the day 8 survey through the app.

Eight days following their vaccination, mobile app participants who activated their accounts received a push notification on their phone to complete their survey. A reminder push notice was sent out on day 11 to participants who had not yet competed the day 8 survey. On November 16, 2020 (midway through the recruitment period), additional email reminders in the mobile app arm were implemented on days 2, 4, and 6 to remind participants to register for the app. All participants received a day 8 email directing them to use their CANImmunize account to complete their influenza vaccine survey. Access to the survey link also was available in the email reminder.

### Usability Survey

Following completion of the safety survey, all participants were sent a separate link via email to complete a user experience survey. Participants were asked about their history of participating in the flu vaccine safety survey and whether they previously used the CANImmunize app. Using a Likert scale, participants were asked questions on (1) perceived ease of use, (2) perceived usefulness, (3) their attitudes and intention of use toward the platform, and (4) questions pertaining to vaccine confidence and safety.

### Statistical Analysis

The data were analyzed within the intention-to-treat arm to which participants were randomized. The data were summarized descriptively. Differences in response rates were compared using a chi-square test. AEFI incidence was compared between groups using a chi-square test and presented with 95% CIs. Response time between groups was assessed using a Student *t* test. A Mann-Whitney test with adjustments for multiple comparisons was used to compare useability responses among new and previous web-based notification platform users. Significance was accepted as *P*<.05.

### Ethical Considerations

All participants provided informed electronic consent upon completion of the web-based survey for primary data collection as well as secondary analyses of research data. The Ottawa Health Science Network Research Ethics Board (20200591-01H) and the BC Children’s Hospital Research Ethics Board approved this study. This was a substudy under the CANVAS protocol (OHSN REB: 20100715-01H, BC: H10-02274). All participants’ personal data were anonymized to protect their privacy and confidentiality. Participants did not receive any monetary compensation for participating in this study.

## Results

### Overview

**Figure 1:**
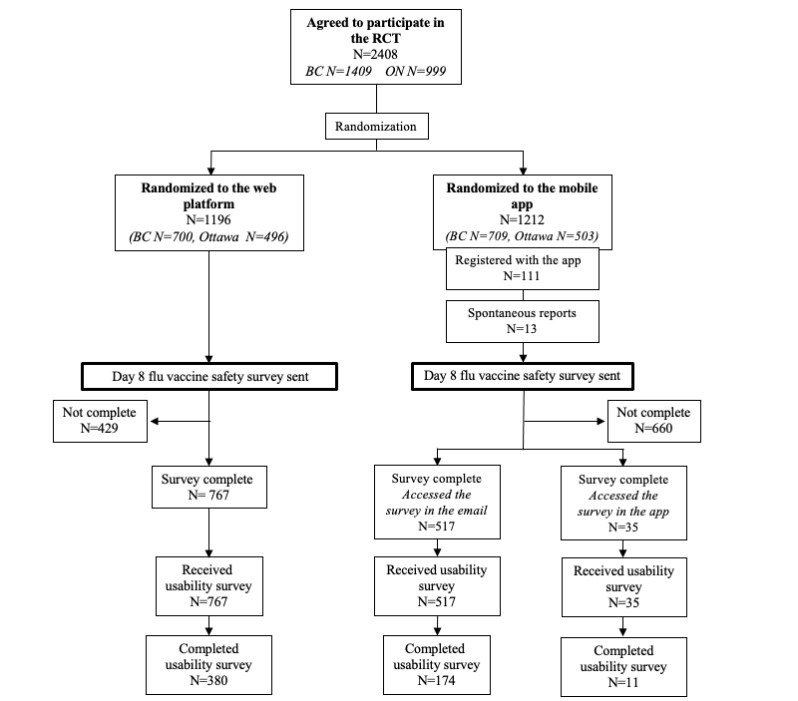
Overview of participant recruitment, randomization, and survey completion. RCT: randomized controlled trial.

Between October 6 and November 29, 2020, a total of 2408 individuals agreed to participate in the RCT (Vancouver, n=1409; Ottawa, n=999). In total, 1196 participants were randomized to the CANVAS arm and 1212 to the CANImmunize arm (Figure 1).

Overall, 1319 (54%) RCT participants completed the web-based safety survey. Participant demographics are presented in Table 1. The completion rate was higher among web-based notification platform users (64%, n=767) than among mobile app users (45%, n=552; *P*<.001).

Of the 552 mobile app respondents, 15% (N=87) created a CANImmunize account and 4.3% (n=24) already had an account, 35 (6.3%) accessed the survey using the app, and the remaining participants (n=517, 93.6%) accessed the survey in the reminder email.

Of the users who created a new account, 100 (90% of registered users) registered for their account after November 16, 2020, compared to 10 before. Initiation of the reminder emails improved survey completion in the CANImmunize arm, with 73% (n=404) of mobile app survey completions occurring after the implementation of additional reminder emails.

**Table 1:** Demographic details of participants (N=1319) who submitted a flu vaccine safety survey (intention-to-treat arm).

		Web-based notification (n=767), n (%)	Mobile app (n=552), n (%)	Total, n (%)
**Sex**				
	Female	430 (56.1)	325 (58.9)	755 (57.2)
	Male	333 (43.4)	226 (40.9)	559 (42.4)
	Other	4 (0.5)	1 (0.2)	5 (0.4)
**Age group**				
	6-23 months	36 (4.7)	28 (5.1)	64 (4.8)
	2-4 years	87 (11.3)	81 (14.7)	168 (12.7)
	5-9 years	138 (18.0)	74 (13.4)	212 (16.1)
	10-14 years	96 (12.5)	66 (11.9)	162 (12.3)
	15-19 years	23 (3.0)	14 (2.5)	37 (2.8)
	20-29 years	43 (5.6)	32 (5.8)	75 (5.7)
	30-39 years	120 (15.6)	114 (20.6)	234 (17.7)
	40-49 years	142 (18.5)	83 (15.0)	225 (17.1)
	50-64 years	76 (9.9)	55 (10.0)	131 (9.9)
	65-79 years	5 (0.6)	4 (0.7)	9 (0.7)
	80+ years	1 (0.1)	1 (0.2)	2 (0.2)
**Number of Flu vaccines in the last 2 years**				
	0	78 (10.2)	53 (9.6)	131 (11.7)
	1	141 (18.4)	100 (18.1)	241 (3.7)
	2	548 (71.4)	399 (72.3)	947 (84.6)
**Previous** **web-based notification platform** **usage**				
	Yes	171 (44.4)^a^	78 (40.2)^b^	249 (43.0)^c^
	No	198 (51.4)^a^	98 (50.5)^b^	296 (51.1)^c^
	Unknown	16 (4.2)^a^	18 (9.3)^b^	34 (5.9)^c^
**Existing app users**				
	Yes	N/A^d^	26 (13.4)	N/A
	No	N/A	158 (81.4)	N/A
	Unknown	N/A	10 (5.2)	N/A

^a^Calculated on the basis of a total of 385 participants.

^b^Calculated on the basis of a total of 194 participants.

^c^Calculated on the basis of a total of 579 participants.

^d^N/A: not applicable.

### Response Time

The mean response time for the day 8 survey was slightly longer for the mobile app group than for the web-based notification platform group (10.7, SD 3.9 days vs 10.1, SD 3.2 days, respectively; *P*=.001).

### Spontaneous Reports

Spontaneous reports were available for mobile app users only. In total, 27 mobile app users accessed the spontaneous report survey, and 13 users submitted a spontaneous report, 1 of which was considered incomplete. The spontaneous reports were submitted on average 4.3 (SD 1.3, median 4) days after vacation. One of the 13 spontaneous reports was medically attended. Seven of the completed reports did not indicate any symptoms, suggesting that participants may have submitted their day 8 survey early or may have misunderstood the spontaneous report since they did not report any AEFIs despite the purpose of the survey being to gather AEFI information.

### Event Reporting

Approximately 10% (n=134) of study participants reported experiencing a new or worsening health problem (78 web-based notification system users vs 56 mobile app users). In total, 43 of these participants (29 web-based notification system users vs 14 mobile app users) reported missing work or activities or consulting a medical professional (Table 2). No significant differences were observed in incidence rates between web-based and mobile app participants. The relative risk of developing or worsening of any health problem for the web-based notification arm relative to those in the mobile app arm was 1.002 (95% CI 0.7226-1.39). The relative risk of developing or worsening of any nonsevere health problem was 0.839 (95% CI 0.5702-1.2649). The relative risk of a health problem being severe enough to miss work or activities or a medical consultation was 1.491 (95% CI 0.7855-2.7619).

**Table 2:** Adverse event reporting rates among participants.

	Web-based notification system users, n (rate, 95% CI)	Mobile app users, n (rate, 95% CI)
Development or worsening of any health problem	78 (10.17, 8.12-12.53)	56 (10.14, 7.8-13.0)
Development or worsening of any nonsevere health problem	49 (6.39, 4.76-8.36)	42 (7.61, 5.5-10.1)
Adverse event severe enough to miss work or activities or a medical consultation	29 (3.78, 2.55-5.39)	14 (2.54, 1.4-4.2)

### Usability and Perceived Usefulness

In total, 579 participants took the usability survey, with 194 mobile app and 385 web-based notification respondents, respectively. In total, 249 of those completing the usability survey had previously participated in the influenza vaccine safety survey (n=249, 43% of usability respondents) and 13% of usability respondents had used CANImmunize before (mobile app arm only, n=26).

Of the 35 mobile app users who completed the survey in the app, only 11 completed the useability survey; hence, we have only reported on the usability responses of the web-based notification platform users (Table 3). We conducted a sensitivity analysis among existing and new web platform users. There was no difference in responses between new and previous users of the web-based notification survey respondents (Multimedia Appendix 1).

Overall, 99% of web-based notification platform users agreed or strongly agreed that the platform was easy to use, and 88.8% of them thought that it made reporting AEFI easier.

When asked about perceived usefulness, 73.8% of web-based notification platform users agreed or strongly agreed that it will make vaccines safer, and 91.4% of them agreed or strongly agreed that it could make it easier for public health professionals to detect vaccine safety issues. The majority of participants (88.8%) agreed or strongly agreed that it made reporting a vaccine side effect easier, using this system was a good idea (93.2%), and that they would use it for additional vaccines (85.9%) or the COVID-19 vaccine (94.0%). Despite this, only 47.4% of web-based notification platform users who completed the usability survey reported that it increased their confidence in vaccine safety. Only 77.7% of users felt confident about data privacy and security.

**Table 3:** Perceived ease of use, usefulness, attitudes toward use, and intention to use among web-based notification survey users.

	Web-based notification survey (n=385)					
	Total respondents, n	Strongly disagree, n (%)	Disagree, n (%)	Neither agree nor disagree, n (%)	Agree, n (%)	Strongly agree, n (%)
Easy to use	380	1 (0.3)	0 (0)	2 (1.1)	61 (33.2)	316 (83.2)
Easy to open	382	1 (0.3)	0 (0)	2 (1.1)	75 (40.8)	304 (79.6)
Easy to access	377	0 (0)	0 (0)	2 (1.1)	72 (39.1)	303 (80.4)
This system will help make vaccines safer	382	2 (0.5)	3 (0.8)	95 (24.9)	132 (34.6)	150 (39.3)
This system increased my awareness of vaccine records	377	11 (2.9)	45 (11.9)	155 (41.1)	80 (21.2)	86 (22.8)
This system could make it easier for public health to detect safety issues with new vaccines	382	1 (0.3)	0 (0)	32 (8.4)	181 (47.4)	168 (44)
This system allows me to easily report a vaccine side effect (an adverse event) following immunization	376	5 (1.3)	3 (0.8)	34 (9.0)	150 (39.9)	184 (48.9)
Using this system to report vaccine side effects (adverse events following immunization) is a good idea	380	1 (0.3)	2 (0.5)	23 (6.1)	144 (37.9)	210 (55.3)
I feel confident about the privacy and security of my data in this system	381	1 (0.3)	3 (0.8)	81 (21.3)	152 (39.9)	144 (37.8)
If this was available for additional vaccines, I would use it	382	1 (0.3)­	1 (0.3)	52 (13.6)	142 (37.2)	186 (48.7)
I would use this system for a new COVID-19 vaccine	382	1 (0.3)	0 (0)	22 (5.8)	142 (37.2)	217 (56.8)
This system increased my confidence in the safety of vaccines	378	1 (0.3)	19 (5.0)	179 (47.4)	87 (23.0)	92 (24.3)
Getting vaccinated is a good way to protect myself and my family from disease	374	1 (0.3)	0 (0)	6 (1.6)	71 (19.0)	296 (79.1)
Vaccinating myself and my family is important for the health of others in my community	379	1 (0.3)	0 (0)	3 (0.8)	68 (17.9)	307 (81)
I am concerned about serious side effects of vaccines	381	32 (8.4)	94 (24.7)	80 (21.0)	106 (27.8)	69 (18.1)
New vaccines carry more risks than older vaccines	383	31 (8.1)	41 (10.7)	187 (48.8)	85 (22.2)	39 (10.2)

## Discussion

### Principal Findings

This study compared 2 digital reporting systems (a web-based notification platform vs a mobile app) for AEFI reporting during the 2020 seasonal influenza vaccination campaign. In total, 2408 individuals agreed to participate in the RCT. A total of 1196 participants were randomized to the CANVAS arm and 1212 to the CANImmunize arm (Figure 1). Overall, 1319 (54%) RCT participants completed the web-based safety survey. The completion rate was higher among web-based notification platform users (64%, n=767) than among mobile app users (45%, n=552). Ease of use was higher among web-based notification platform users. Our findings suggest that a web-based survey link is an acceptable approach for active, participant-centered AEFI reporting. We identified a number of concerns with mobile app reporting, which would need to be addressed to improve the acceptability and usability of app-based reporting.

To be successful, digital solutions must be easy to use, easy to access, and meet the needs of the users [14]. In this study, we saw lower rates of participant engagement in the mobile app arm and were unable to assess usability due to low participant responses. Participants were required to download the app and register their account. This added complexity and was likely a barrier to use.

Interestingly, the highest response rate in the mobile app group was observed among 30-39–year-old participants. This was possibly due to this demographic being having a higher level of technological literacy and comfort with mobile apps [15]. The lowest response rate among the mobile app users was observed among older adults aged >65 years. The low response rate among older adults may be due to less comfort with mobile apps or possibly barriers such as difficulty reading smaller font [16].

The lack of responses to the usability survey is also a potential concern as we were unable to assess exactly what needs to change to make mobile app reporting acceptable and user-friendly. The usability survey was provided as a separate email link after the safety survey was completed, and it may have been missed by users.

It is critical to make mHealth solutions accessible, and a barrier may be technological literacy or low perceived ease of use among some populations. It is important that mHealth apps are designed to be user-friendly for target demographics [17]. This is important in vaccine safety surveillance studies that need to capture data from a breadth of demographics.

Current literature suggests that the development of mHealth apps should follow 8 specific categories [17] with usability being one of the key categories. Usability refers to the app being adapted to the target population [17]. Making the app easy to use, with clear instructions, and the feedback of various community members who use the app is critical. Our finding—a lower response rate was observed among mobile app users than among the web-based notification platform users—implies that the mobile app may not have been optimally designed for usability for all of the target demographics. The app was possibly challenging to navigate or perceived to be so resulting in a lower response rate than that among users of the web-based notification system.

Enabling AEFI reporting from individuals who are already using a mobile app for another purpose would require less work for the user and may be a more successful approach. For example, 42% of Canadians report accessing websites, mobile apps, or other interactive web-based services to support or monitor their health [18]. Integrating use with these types of health monitoring apps would be an approach worth trialing. Indeed, since the completion of this study, the CANVAS-COVID safety survey has recruited control participants from among current CANImmunize users [19]. Other digital technologies have been used during the COVID-19 vaccine rollout. Social media was used to collect postmarket vaccine safety data [20-23], and the US Centers for Disease Control and Prevention created the V-safe After Vaccination Health Checker application, which allowed individuals to register and complete vaccine safety surveys after receiving their COVID-19 vaccine [23].

Based on the RCT conducted in this study, it was found that adding the extra step of using the mobile app introduced an additional barrier for participants who may have lower technological literacy. Future mHealth developments should include various participants from the target demographics in the development process to ensure usability.

### Limitations

This study has several limitations. Mobile app users were required to register for a CANImmunize account for the study. Registration allowed for influenza vaccine data to be uploaded to the users’ CANImmunize account; however, this additional step appeared to be a barrier to use as we saw low registration numbers. Study participants were randomized after study registration; hence, onsite personnel were not able to support participants randomized to the mobile app arm in activating their account, which may also have contributed to low account registration. However, this can be perceived as a strength as this provided more real-world effectiveness data of app usage as in a nonstudy setting, users would have to navigate this on their own without study staff assistance. The day 8 survey email served as a reminder to mobile app users to register their accounts if they had not yet done so, but it also provided a direct link to the web-based safety and usability surveys, which 94% of mobile app arm users who completed the survey chose to use, rather than registering for a mobile app account. As a result, we could not effectively evaluate user preference or experiences in the mobile app arm of the study. Another major limitation is that data about nonresponders were not collected, which could introduce risk for response bias, particularly since the response rate differed between the web-based notification arm (64%) and the mobile app arm (45%).

### Conclusions

In summary, this study demonstrated high user acceptability with the web-based survey platform compared to that with a mobile app. Making AEFI reporting available to existing mobile app users may still be a viable initiative for participant-centered active reporting in Canada but would require further refinement.

## Appendix

Multimedia Appendix 1 Useability survey responses for new compared to previous users of the influenza vaccine safety survey web platform.

Multimedia Appendix 2 CONSORT-eHEALTH checklist (V 1.6.1).

## Abbreviations

AE: adverse event
AEFI: adverse event following immunization
CANVAS: Canadian National Vaccine Safety Network
mHealth: mobile health
RCT: randomized controlled trial

## Data Availability

Data are available upon request.
