# Supplementary material for: Comparing the Use of a Mobile App and a Web-Based Notification Platform for Surveillance of Adverse Events Following Influenza Immunization: Randomized Controlled Trial
Source: JMIR Public Health Surveill. 2023 May 8;9:e39700. doi: 10.2196/39700 (PMC10203918; doi:10.2196/39700)
Supplement: Multimedia Appendix 1 [file publichealth_v9i1e39700_app1.docx]

Useability survey responses for new compared to previous users of the influenza vaccine safety survey web platform

|  | **Previous Users (N=171)** | | | | | | **New Users (N=214)** | | | | | | **FDR corrected p value** |
| --- | --- | --- | --- | --- | --- | --- | --- | --- | --- | --- | --- | --- | --- |
|  | **Total number of respondents (n)** | **% Strongly Disagree**  **(n)** | **% Disagree**  **(n)** | **% Neither Agree nor Disagree (n)** | **% Agree (n)** | **% Strongly Agree (n)** | **Total number of respondents (n)** | **% Strongly Disagree**  **(n)** | **% Disagree**  **(n)** | **% Neither Agree nor Disagree (n)** | **% Agree (n)** | **% Strongly Agree (n)** | **0.647** |
| Easy to use | 168 | 0.6% (1) | 0.0% (0) | 1.2% (2) | 12.5% (21) | 85.7% (144) | 212 | 0.0% (0) | 0.0% (0) | 0.0% (0) | 18.9% (40) | 81.8% (172) | 0.647 |
| Easy to open | 170 | 0.6% (1) | 0.0% (0) | 1.2% (2) | 15.9% (27) | 82.4% (140) | 212 | 0.0% (0) | 0.0% (0) | 0.0% (0) | 22.6% (48) | 77.4% (164) | 0.647 |
| Easy to access | 170 | 0.0% (0) | 0.0% (0) | 1.2% (2) | 15.9% (27) | 82.9% (141) | 207 | 0.0% (0) | 0.0% (0) | 0.0% (0) | 21.7% (45) | 78.3% (162) | 0.647 |
| This system will help make vaccines safer | 171 | 0.6% (1) | 1.8% (3) | 26.3% (45) | 32.2% (55) | 39.2% (67) | 211 | 0.5% (1) | 0.0% (0) | 23.7% (50) | 36.5% (77) | 39.3% (83) | 0.653 |
| This system increased my awareness of vaccine records | 167 | 3.0% (5) | 15.0% (25) | 40.1% (67) | 19.2% (32) | 22.8% (38) | 210 | 2.9% (6) | 9.5% (20) | 41.9% (88) | 22.9% (48) | 22.9% (48) | 0.653 |
| This system could make it easier for public health to detect safety issues with new vaccines | 171 | 0.6% (1) | 0.0% (0) | 11.1% (19) | 39.8% (68) | 48.5% (83) | 211 | 0.0% (0) | 0.0% (0) | 6.2% (13) | 53.6% (113) | 40.3% (85) | 0.653 |
| This system allows me to easily report a vaccine side effect (an adverse event) following immunization | 171 | 0.6% (1) | 0.0% (0) | 12.9% (22) | 27.5% (47) | 59.1% (101) | 205 | 2.0% (4) | 1.5% (3) | 5.9% (12) | 50.2% (103) | 40.5% (83) | 0.082 |
| Using this system to report vaccine side effects (adverse events) AEFIs is a good idea | 168 | 0.6% (1) | 0.0% (0) | 10.1% (17) | 30.4% (51) | 58.9% (99) | 212 | 0.0% (0) | 0.9% (2) | 2.8% (6) | 43.9% (93) | 52.4% (111) | 0.653 |
| I feel confident about the privacy and security of my data in this system | 171 | 0.6% (1) | 1.8% (3) | 25.7% (44) | 34.5% (59) | 37.4% (64) | 210 | 0.0% (0) | 0.0% (0) | 17.6% (37) | 44.3% (93) | 38.1% (80) | 0.647 |
| If this was available for additional vaccines, I would use it | 171 | 0.6% (1) | 0.0% (0) | 13.5% (23) | 34.5% (59) | 51.5% (88) | 211 | 0.0% (0) | 0.5% (1) | 13.7% (29) | 39.3% (83) | 46.4% (98) | 0.653 |
| I would use this system for a new COVID-19 vaccine | 171 | 0.6% (1) | 0.0% (0) | 5.3% (9) | 35.7% (61) | 58.5% (100) | 211 | 0.0% (0) | 0.0% (0) | 6.2% (13) | 38.4% (81) | 55.5% (117) | 0.653 |
| This system increased my confidence in the safety of vaccines | 167 | 0.6% (1) | 6.6% (11) | 46.1% (77) | 19.8% (33) | 26.9% (45) | 211 | 0.0% (0) | 3.8% (8) | 48.3% (102) | 25.6% (54) | 22.3% (47) | 0.941 |
| Getting vaccinated is a good way to protect myself and my family from disease | 166 | 0.6% (1) | 0.0% (0) | 1.2% (2) | 14.5% (24) | 83.7% (139) | 208 | 0.0% (0) | 0.0% (0) | 1.9% (4) | 22.6% (47) | 75.5% (157) | 0.298 |
| Vaccinating myself and my family is important for the health of others in my community | 167 | 0.6% (1) | 0.0% (0) | 0.0% (0) | 12.6% (21) | 86.8% (145) | 212 | 0.0% (0) | 0.0% (0) | 1.4% (3) | 22.2% (47) | 76.4% (162) | 0.082 |
| I am concerned about serious side effects of vaccines | 169 | 8.3% (14) | 26.0% (44) | 19.5% (33) | 31.4% (53) | 14.8% (25) | 212 | 8.5% (18) | 23.6% (50) | 22.2% (47) | 25.0% (53) | 20.8% (44) | 0.653 |
| New vaccines carry more risks than older vaccines | 171 | 7.6% (13) | 14.6% (25) | 41.5% (71) | 26.3% (45) | 9.9% (17) | 212 | 8.5% (18) | 7.5% (16) | 54.7% (116) | 18.9% (40) | 10.4% (22) | 0.865 |
